# Does Multifunctional Acrylate’s Addition to Methacrylate Improve Its Flexural Properties and Bond Ability to CAD/CAM PMMA Block?

**DOI:** 10.3390/ma15217564

**Published:** 2022-10-28

**Authors:** Yukinori Maruo, Kumiko Yoshihara, Masao Irie, Noriyuki Nagaoka, Takuya Matsumoto, Shogo Minagi

**Affiliations:** 1 Department of Prosthodontics, Okayama University, 2-5-1 Shikata-cho, Okayama 700-8525, Japan; 2 Health Research Institute, National Institute of Advanced Industrial Science and Technology, 2217-14 Hayashi-cho, Takamatsu, Kagawa 761-0395, Japan; 3 Department of Biomaterials, Okayama University Graduate School of Medicine, Dentistry and Pharmaceutical Sciences, 2-5-1 Shikata-cho, Okayama 700-8525, Japan; 4 Advanced Research Center for Oral and Craniofacial Sciences, Okayama University Dental School, Okayama 700-8558, Japan

**Keywords:** acrylate, methacrylate, CAD/CAM, flexural strength, shear bond strength

## Abstract

This study investigated the effects of a multifunctional acrylate copolymer—Trimethylolpropane Triacrylate (TMPTA) and Di-pentaerythritol Polyacrylate (A-DPH)—on the mechanical properties of chemically polymerized acrylic resin and its bond strength to a CAD/CAM polymethyl methacrylate (PMMA) disk. The methyl methacrylate (MMA) samples were doped with one of the following comonomers: TMPTA, A-DPH, or Trimethylolpropane Trimethacrylate (TMPTMA). The doping ratio ranged from 10 wt% to 50 wt% in 10 wt% increments. The flexural strength (FS) and modulus (FM) of PMMA with and without comonomer doping, as well as the shear bond strength (SBS) between the comonomer-doped PMMA and CAD/CAM PMMA disk, were evaluated. The highest FS (93.2 ± 4.2 MPa) was obtained when doped with 20 wt% of TMPTA. For TMPTMA, the FS decreased with the increase in the doping ratio. For SBS, TMPTA showed almost constant values (ranging from 7.0 to 8.2 MPa) regardless of the doping amount, and A-DPH peaked at 10 wt% doping (8.7 ± 2.2 MPa). TMPTMA showed two peaks at 10 wt% (7.2 ± 2.6 MPa) and 40 wt% (6.5 ± 2.3 MPa). Regarding the failure mode, TMPTMA showed mostly adhesive failure between the CAD/CAM PMMA disk and acrylic resin while TMPTA and A-DPH showed an increased rate of cohesive or mixed failures. Acrylate’s addition as a comonomer to PMMA provided improved mechanical properties and bond strength to the CAD/CAM PMMA disk.

## 1. Introduction

CAD/CAM technology and metal-free materials are widely used in esthetic dentistry [1]. As CAD/CAM-fabricated-metal-free restorations meet the esthetic needs of patients and do not trigger allergic reactions [2], the use of CAD/CAM technology has extended beyond conservative restorations and fixed prostheses to removable prostheses, and from wax patterns to the manufacturing of frameworks or retainers of prostheses using metals and ceramics [3,4]. For complete dentures, CAD/CAM technology coupled with intraoral scanners have been used to fabricate both the custom tray and denture, thereby reducing the complexity of the technical procedure and the inherent technical errors [5,6,7].

Acrylic-based polymethyl methacrylate (PMMA) resins are conventionally used as the occlusal record or denture base material. However, their shortcomings include high shrinkage, water absorption, and low wear resistance. The large polymerization shrinkage that occurs during the record base’s fabrication can aggravate occlusal errors and adversely affect the fit with the mucosal surface [8]. Polymerization shrinkage, which occurs during the final step of the denture base’s fabrication, can alter occlusal contact integrity and necessitate a long chairside time for the base’s adjustment. Although various polymerization procedures have been introduced to reduce this shrinkage, it cannot be completely eliminated [9].

To overcome polymerization shrinkage, CAD/CAM PMMA disks have been introduced as the material of choice for denture bases because they are manufactured under controlled, standardized, industrial conditions of high pressure and temperature [10,11]. CAD/CAM disks with multilayer tooth colors are also commercially available. It has been reported that well-fitting dentures with accurate occlusion were obtained when both the denture base and artificial teeth were milled from PMMA or composite CAD/CAM disks [12].

To achieve a natural gingival margin, denture teeth must be bonded onto the tooth sockets provided by a milled-denture base [13,14]. Compared to heat-polymerized PMMA, as a denture base material, CAD/CAM PMMA disks offer better physical properties and lower wear resistance because of their improved conversion and reduced residual monomer content. However, the lower unpolymerized content of CAD/CAM PMMA disks lowers the bond strength between artificial teeth and the denture base, thereby increasing the risk of the detachment of the artificial teeth [15,16,17,18].

To overcome the limitations of chemically autopolymerized acrylic resins, chemical modifications with the addition of a copolymer have been introduced to improve flexural strength, flexural modulus, impact strength, thermal durability, and adhesion properties. This is achieved by increasing the crosslinking rate among the polymer beads and the polymerization rate [19,20]. The type of copolymer and the number of crosslinking agents in the polymer have significant effects on their mechanical properties [21]. Multifunctional methacrylates such as fluoroalkyl methacrylate, butadiene styrene, 2-hydroxyethyl methacrylate, isobutyl methacrylate, 1,3-bis(methacryloxypropyl) tetramethyldisiloxane, narbonyl, and phenyl methacrylate monomers have been treated with MMA and used to enhance the mechanical and physical properties of acrylic resins.

In general, bifunctional methacrylates or acrylates are added to acrylic resins. However, bifunctional methacrylates have a small molecular framework and low flexibility, which leads to poor double bond reactivity and results in a large number of unreacted double bonds. In contrast, acrylates possess high flexibility, excellent adhesion, low skin irritation properties, and outstanding water resistance. Hence, they are widely used for coating materials, plastic films, and flooring materials [22]. A multifunctional acrylate is an acrylate ester consisting of several acrylic groups in the molecule. It is widely used in light-cured and heat-cured resins [23]. These monomers consist of electron-rich molecules, and molecular polarity affects their solubility in resins as well as their mechanical properties. Further, acrylates polymerize faster than methacrylates, which accounts for the high mechanical properties of acrylate polymers [22].

The aims of this study were to investigate whether the copolymeric addition of trimethylolpropane triacrylate (TMPTA), di-pentaerythritol polyacrylate (A-DPH), and trimethylolpropane trimethacrylate (TMPTMA) to MMA would affect the: (1) mechanical properties of the chemically polymerized acrylic resin and (2) the bond strength to the CAD/CAM PMMA disk. The null hypotheses tested in this study were as follows: (1) the addition of an acrylate monomer to MMA would not affect the mechanical properties of the chemically polymerized acrylic resin, and (2) there would be no differences in the bond ability to CAD/CAM PMMA disk.

## 2. Materials and Methods

### 2.1. Materials Preparation

Methyl methacrylate (MMA; Mitsubishi Chemical, Tokyo, Japan) was used as the main monomer. Each comonomer, either a methacrylate or acrylate monomer, was added to enhance the crosslinking structure (Table 1). Mixing ratios for monomer liquid of MMA, comonomers, and polymerization initiator of dimethyl-p-toluidine (DMPT, Fujifilm Wako Pure Chemical, Osaka, Japan) are shown in Table 2, ranging from 0 to 50 wt% in 10 wt% increments.

The methacrylate comonomer used for doping was Trimethylolpropane Trimethacrylate (TMPTMA; Shin-Nakamura Chemical, Wakayama, Japan). Acrylate comonomers were Trimethylolpropane Triacrylate (TMPTA; Shin-Nakamura Chemical) and Di-pentaerythritol Polyacrylate (A-DPH; Shin-Nakamura Chemical). Mixed monomer liquid material was stirred for 24 h and stored.

### 2.2. Static Three-Point Flexural Test

The PMMA polymer consisting of copolymers of methacrylic esters, benzoyl peroxide, and others (Metafast, Sun Medical, Shiga, Japan), as well as the prepared monomer liquid, were mixed in 3:2 polymer-monomer weight ratio for 10 s. Mixed material was filled into a Teflon mold (2 × 2 × 25 mm), pressed under 5 N load for 10 min, and polymerized in a pressure-curing unit (SSKJ-50, Shofu Co., Kyoto, Japan) at 50 °C and approximately 0.4 MPa pressure for 10 min to stipulate the polymerization condition. After final polishing, bar-shaped samples (*n* = 10 for each group) were stored in distilled water for 24 h.

**Figure 1:**
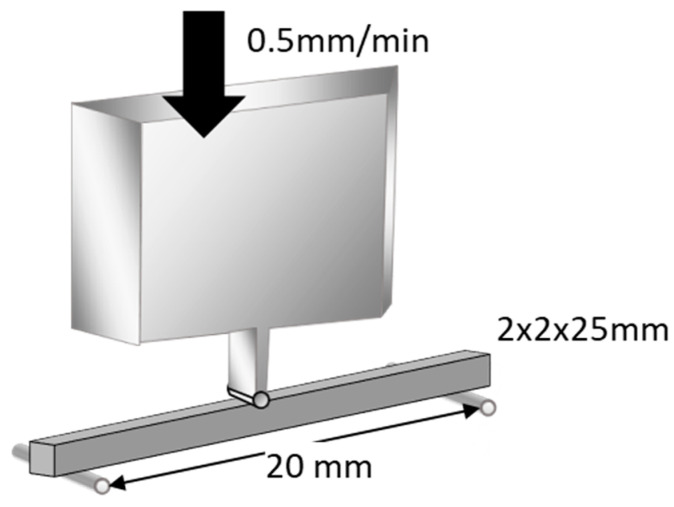
Schematic illustration of flexural strength test. Flexural strength was measured using a three-point-bending technique with a 20 mm span and a crosshead speed of 0.5 mm/min (reproduced with permission from Maruo Y. et al. Acta Odontol. Scand. 2015 [24]).

Flexural strength was measured using a three-point bending test over a 20 mm span at a crosshead speed of 0.5 mm/min (Model 5565, Instron, Norwood, MA, USA), as outlined in ISO 9917-2 (Figure 1). An external force of maximum 5 kgf (49 N) was applied to its midsection until fracture occurred. Flexural strength and flexural modulus were automatically calculated using a bundled software. The data were subjected to Levene’s test to evaluate homogeneity of variance (*p* < 0.05) and were statistically compared using one-way analysis of variance (ANOVA) and Tukey’s post hoc test within 5% error limits (*p* < 0.05) using the Statistical Package for the Social Sciences (IBM SPSS Statistics).

### 2.3. Shear Bond Strength Test

**Figure 2:**
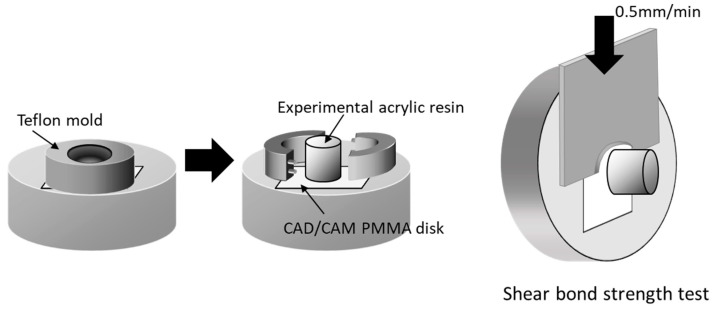
Schematic illustration of shear bond strength test. Acrylic resin column was cemented onto PMMA surface with a Teflon jig mold. Shear bond strength was measured using a universal testing machine with a crosshead speed of 0.5 mm/min (reproduced with permission from Maruo Y. et al. J. Appl. Biomater. Funct. Mater. 2017 [25]).

PMMA disk-shaped specimens (M-PM^®^ Disc, Merz Dental GmbH, Germany) were polished with abrasive paper (Silicon Carbide abrasive paper, #2000, Struers A/S, Rodovre, Denmark) under water irrigation to preclude any mechanical bonding. PMMA polymer and prepared monomer liquid were mixed at a 3:2 polymer–monomer weight ratio. This mixture was poured to fill the matrix on the test surface of all specimens to form a resin column with a 2 mm thickness and 3.6 mm diameter (Figure 2). Resin polymerization was performed in a pressure-curing unit (SSKJ-50, Shofu Co., Kyoto, Japan) at 50 °C and approximately 0.4 MPa pressure for 10 min to stipulate the polymerization condition. All bonded specimens were immersed in distilled water (37 ± 2 °C) for 24 h before shear bond strength test.

Each specimen was placed in a shear test fixture, and shear bond strength was measured using a universal testing machine (Autograph AG-X, Shimadzu, Japan) at a crosshead speed of 0.5 mm/min (Autograph AG-X, Shimazu, Kyoto, Japan)—as outlined in ISO 29,022—with a notched edge shear blade (Figure 2). Stress at failure was automatically calculated and recorded as the shear bond strength using bundled software. After test, the fractured surfaces were examined under a light microscope to basically ascertain the nature of fractures [26]. Failure caused by shear fracture was classified into one of the following three types: adhesive failure, cohesive failure, or mixed-mode failure (adhesive–cohesive). Shear bond strength data of each group were subjected to Levene’s test to evaluate homogeneity of variance (*p* < 0.05) and were statistically compared using one-way analysis of variance (ANOVA) and Tukey’s post hoc test within 5% error limits (*p* < 0.05) using the Statistical Package for the Social Sciences (IBM SPSS Statistics).

### 2.4. Degree of Conversion

PMMA polymer and prepared monomer liquid were mixed at 3:2 polymer-monomer weight ratio for 10 s. This mixture was crimped onto two potassium bromide (KBr) plates for infrared microscopy (Jasco Engineering Co., Ltd., Tokyo, Japan), and absorbance was measured using a Fourier transform infrared spectrophotometer (IRAffinity-1, Shimadzu Corporation). Using a single-beam method with a measurement range of 500–4000 cm^−1^, 32 integrations, and a resolution of 4.00 cm^−1^, the absorbance of the samples was measured before and after polymerization at 25 °C. Degree of conversion (DC, %) for each group (*n* = 3) was calculated from the absorption peak near 1640 cm^−1^ (which represented the stretching vibration of vinyl group (C=C)) and the absorption peak near 1710 cm^−1^ (which represented the stretching vibration of carbonyl group (C=O)).

## 3. Results

### 3.1. Statis Three-Point Flexural Test

**Figure 3:**
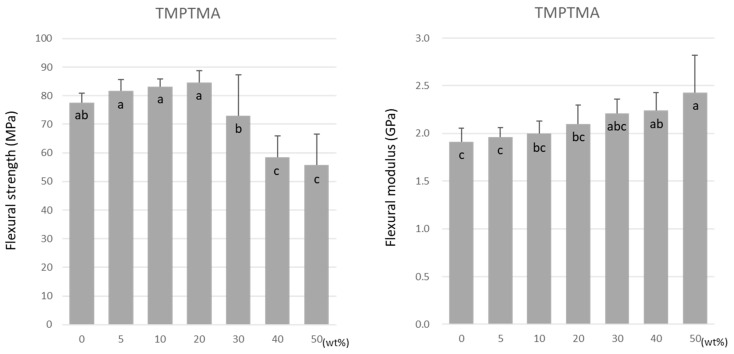
Flexural strength (**left**) and flexural modulus (**right**) of TMPTMA. Same letters (a–c) in each column are not significantly different (*p* > 0.05).

For TMPTMA, the flexural strength was highest at 20 wt% at 84.6 ± 4.1 MPa (Figure 3). The flexural strength significantly lowered as the doping ratio rose above 20 wt%. The lowest value of 55.7 ± 11.0 MPa was obtained at 50 wt% under all experimental conditions. In contrast, the flexural modulus increased significantly with the increase in the doping ratio (Figure 3).

**Figure 4:**
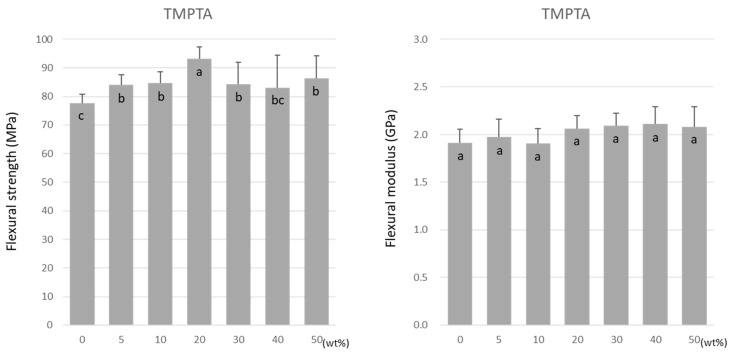
Flexural strength (**left**) and flexural modulus (**right**) of TMPTA. Same letters (a–c) in each column are not significantly different (*p* > 0.05).

Similar to TMPTMA, the flexural strength of TMPTA was highest at 20 wt% at 93.2 ± 4.2 MPa (Figure 4). However, a decrease in the flexural strength did not occur as the doping ratio rose above 20 wt%. Unlike TMPTMA, the flexural modulus of TMPTA was not affected by the doping ratio. The values were maintained at approximately 1.9–2.1 GPa (Figure 4).

**Figure 5:**
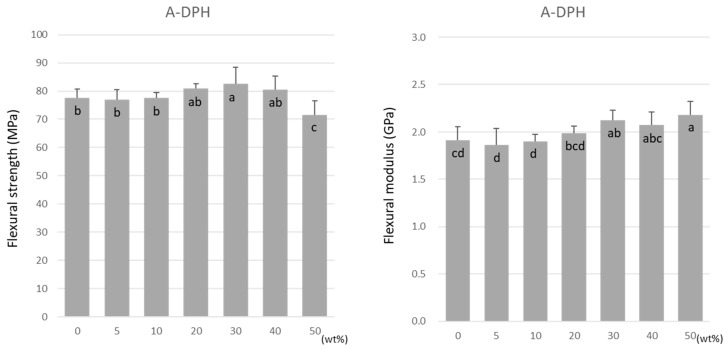
Flexural strength (**left**) and flexural modulus (**right**) of A-DPH. Same letters (a–c) in each column are not significantly different (*p* > 0.05).

For A-DPH, the flexural strength remained constant up to a 40 wt% doping, peaking at 30 wt% at 82.6 ± 5.7 MPa (Figure 5). The flexural strength significantly decreased at 50 wt% but maintained a high value of 71.6 ± 4.9 MPa. Similar to TMPTMA, the flexural modulus increased significantly with the increase in the doping ratio (Figure 5).

### 3.2. Shear Bond Strength Test

**Figure 6:**
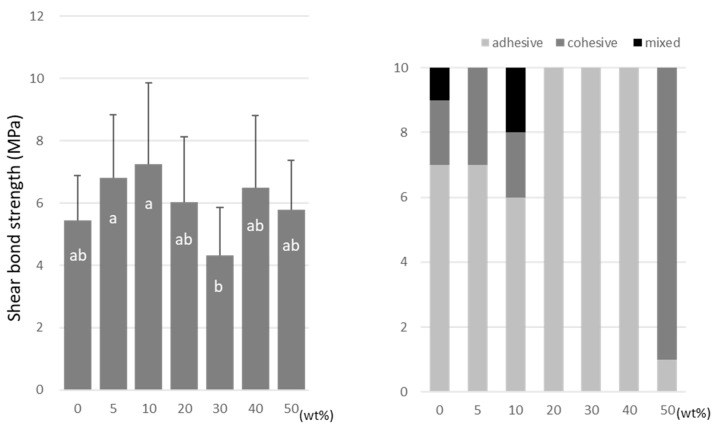
Shear bond strength (**left**) between CAD/CAM PMMA disk and TMPTMA. Same letters (a,b) in each column are not significantly different (*p* > 0.05). Distribution of failure modes after test (**right**).

**Figure 7:**
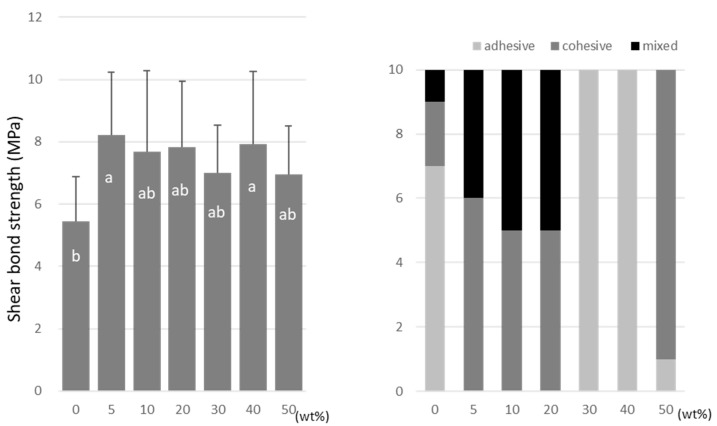
Shear bond strength (**left**) between CAD/CAM PMMA disk and TMPTA. Same letters (a,b) in each column are not significantly different (*p* > 0.05). Distribution of failure modes after test (**right**).

For TMPTMA, the SBS peaked at 10 wt% (7.2 ± 2.6 MPa) and 40 wt% (6.5 ± 2.3 MPa) doping ratios (Figure 6). TMPTA showed almost constant values (ranging from 7.0 to 8.2 MPa) regardless of the doping amount (Figure 7), and the SBS values at 5 wt% (8.2 ± 2.5 MPa) and 40 wt% (7.9 ± 2.9 MPa) were significantly higher than that of the no-doping condition (5.4 ± 1.4 MPa).

**Figure 8:**
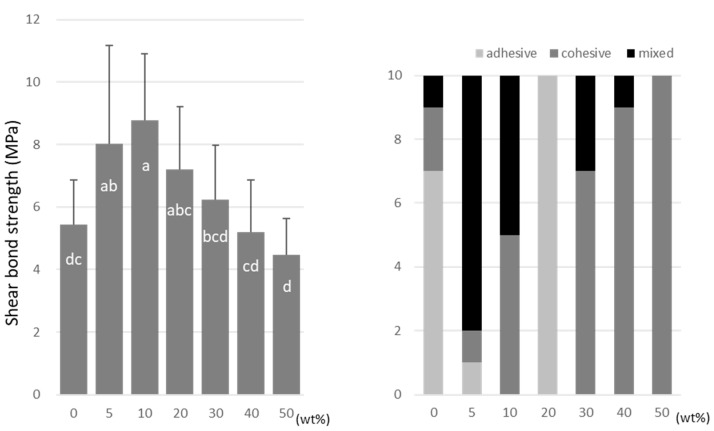
Shear bond strength (**left**) between CAD/CAM PMMA disk and A-DPH. Same letters (a–d) in each column are not significantly different (*p* > 0.05). Distribution of failure modes after test (**right**).

A-DPH peaked at a 10 wt% doping (8.7 ± 2.2 MPa), and the SBS at 50 wt% was significantly lower (4.5 ± 1.1 MPa) than that of the no-doping condition (Figure 8).

TMPTMA showed mostly an adhesive type of failure between the CAD/CAM PMMA disk and resin, while an increased rate of cohesive or mixed failures was detected in TMPTA and A-DPH.

### 3.3. Degree of Conversion

**Figure 9:**
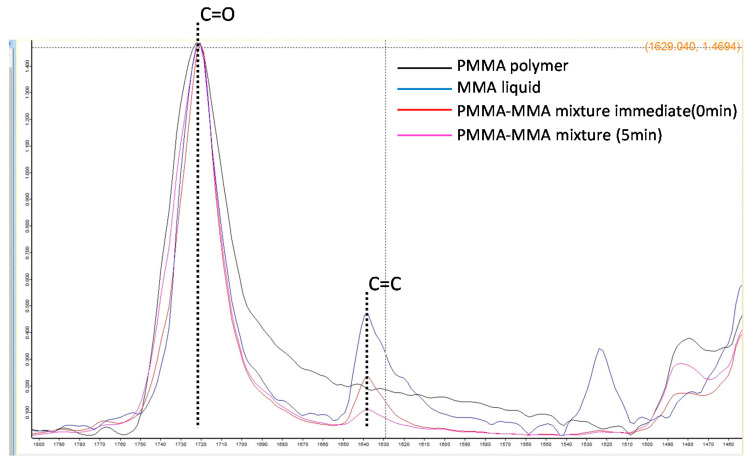
FTIR spectra of the absorption peaks of the vinyl group (C=C) and the carbonyl group (C=O) of each sample.

The DC value of C=O did not change with the comonomer’s addition, while that of C=C decreased with polymerization (Figure 9). The DC values for all comonomers decreased with the increase in the doping ratio (Table 3).

## 4. Discussion

Organic-based resin materials are widely used in the dental field. Cold-cured autopolymerizing polymers are usually supplied as a mixture of PMMA powder beads and an MMA monomer liquid. Crosslinking agents are incorporated as a component of the monomer liquid at the concentration of a few percentages by volume. Multifunctional methacrylates are used as a comonomer to form a crosslinked structure on the long methacrylate main chain. The composition of the comonomers determines the type of three-dimensional polymer network that would be formed after polymerization [27], which is then characterized by the quantity of the remaining double bonds and the crosslinking density [28]. Cross-linkage provides a sufficient number of bridges between linear macromolecules, allowing them to form a three-dimensional network that decreases water sorption, lowers solubility, and increases the strength and rigidity of resin.

The methacrylate group in multifunctional methacrylates has a methyl group attached to the C of the C=C bond, while the acrylate group in the acrylates has a H in this location. The methyl group seems to inhibit the generation of radicals during the polymerization process; hence, this structure would show slower radical polymerization reactivity. Although the less-flexible molecular skeletons of comonomers are also used, they offer lower reactivity of the double C=C bond. As a result, many unpolymerized monomers are trapped in the polymer network. Compared to methacryloyl groups, acryloyl groups have higher radical polymerization reactivity, especially when exposed to light irradiation [29].

Acrylates are widely used as raw materials for paints and photosensitive resins due to their excellent reactivity [30]. The acrylate group, which is a type of vinyl group, has a C=C bond followed by a C=O bond. The C=O bond is a strong bond with a charge distribution or polarity of 779 kJ/mol. The C=O bond promotes the generation of radicals, and this structure shows intense radical polymerization reactivity.

In this study, the following comonomers were added to an MMA monomer: (1) TMPTMA, to serve as a bifunctional methacrylate commonly used as a comonomer; (2) TMPTA, to serve as an acrylate with the same spacer between the functional group and polymerizable group; and (3) A-DPH, to serve as a hexafunctional acrylate monomer with a completely different structure from TMPTMA and TMPTA.

The FS test is used to examine a meaningful mechanical property for resin materials that indicates the stiffness and resistance to fracture during their function. The FM value describes the rigidity of the materials, and materials with a higher FM withstand occlusal forces and preserve the adhesive interface against such forces [31]. The bifunctional methacrylate monomer decreased the FS value but increased the FM value as its doping ratio increased. This could be caused by the lower reactivity of the methacryloyl group compared to the acryloyl group. The C=C double bond of the added comonomers did not polymerize, and three-dimensional crosslinks were not formed completely—eventually, they were detected as a residual monomer in MMA-based resin materials.

The bifunctional acrylate and hexafunctional acrylate did not change the FM value as their doping ratios increased. The FS value also remained high as the doping ratios of the acrylates increased. While acryloyl groups have higher reactivity than methacryloyl groups, the hexafunctional acryloyl monomer presented a reduced improvement in terms of FS compared to that observed with the bifunctional monomer’s addition. The high viscosity and low structural polarity of A-DPH, a hexafunctional monomer, could have resulted in the lower activity of the functional groups compared to the bifunctional monomers. It was probable that the functional groups at close proximity were not fully activated during polymerization, or that the formed polymer structure was a simple one.

Regarding the adhesion to the PMMA CAD/CAM blocks, the latter offered low reactivity because they were already polymerized under industrial conditions of high temperature and high pressure. Doping with each of the three comonomers in this study was shown to increase the adhesive strength to PMMA blocks, whereby the highest SBS was obtained when doped with 10 wt% of A-DPH. This increase in adhesive strength could be attributed to the improvement in mechanical properties or the increase in reactivity brought about by the comonomer’s addition. Notably, A-DPH (a hexafunctional monomer) yielded the highest adhesive strength, probably because of the residual unpolymerized C=C double bond.

TMPTMA with a methacryloyl group and TMPT with an acryloyl group showed a similar biphasic behavior in terms of the SBS values as the doping ratio increased, while the addition of A-DPH showed a monophasic response with a peak at 10 wt% addition. In other words, the comonomer’s addition did not necessarily improve the mechanical properties of acrylic resin even with an increased amount added. Another concern lies with the low water resistance of acrylates. Further studies are needed to examine the water absorption behavior and dissolution rate of acrylates.

The value of the DC decreased with the comonomer’s addition, which would lead to the low mobility of the larger-sized comonomers during polymerization compared to small MMA molecules [32]. In addition, the lower DC value with incurred through the addition of the comonomer could have indicated that more residual comonomers would exist in the resin matrix even after the polymerization process. These residual monomers would cause cytotoxic release in the oral environment during function. Further examinations are needed regarding the residual comonomer release after different periods of water storage. The null hypotheses were not rejected: (1) the acrylate monomer’s addition to MMA did not affect the mechanical properties of the chemically polymerized acrylic resin, and (2) there were no differences in the bond ability to the CAD/CAM PMMA disk.

## 5. Conclusions

Compared to doping with methacrylate, the addition of the acrylate comonomer to the PMMA polymerization offered the polymeric body with improved mechanical properties and an enhanced bond strength to the CAD/CAM disk fabricated from PMMA.

## Figures and Tables

**Table 1:** Structural formula of substances used in this study.

Code	Name	Molecular Weight (g/mol)	Chemical Formula
MMA	Methyl Methacrylate	100.12	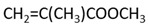
TMPTMA	Trimethylol Propane Trimethacrylate	338.40	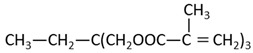
TMPTA	Trimethylol Propane Triacrylate	296.32	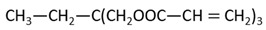
A-DPH	Di-pentaerythritol Polyacrylate	524.51	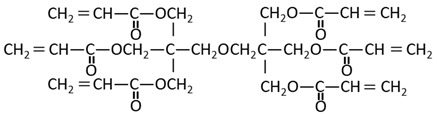

**Table 2:** Composition of samples used in this study.

	Control	10 wt% (mol%)	20 wt% (mol%)	30 wt% (mol%)	40 wt% (mol%)	50 wt% (mol%)
MMA	99	89	79	69	59	49
TMPTMA	-	10 (2.74)	20 (5.07)	30 (6.96)	40 (8.45)	50 (9.52)
TMPTA	-	10 (3.14)	20 (5.83)	30 (8.08)	40 (9.88)	50 (11.23)
A-DPH	-	10 (1.75)	20(3.18)	30(4.31)	40 (5.13)	50 (5.64)
DMPT	1	1	1	1	1	1

**Table 3:** Degree of Conversion (%).

	0	20 wt%	40 wt%
MMA	87.5	-	-
TMPTMA	-	72.3	57.9
TMPTA	-	72.7	67.7
A-DPH		70.4	64.1

## Data Availability

The data presented in this study are available from the corresponding author, Y.M., upon reasonable request.
